# Chromosomal Instability, Aneuploidy, and Cancer

**DOI:** 10.3389/fonc.2014.00161

**Published:** 2014-06-19

**Authors:** Samuel F. Bakhoum, Charles Swanton

**Affiliations:** ^1^Department of Internal Medicine, Mount Auburn Hospital, Cambridge, MA, USA; ^2^Harvard Medical School, Boston, MA, USA; ^3^Cancer Research UK London Research Institute, London, UK; ^4^University College London Cancer Institute, London, UK

**Keywords:** chromosomal instability, aneuploidy, anaphase, chromatin, mitosis, spindle, microtubules, centrosomes

The link between aneuploidy and cancer has been recognized over a century ago (1). Abnormalities in chromosome copy numbers arise from persistent errors in chromosome segregation during cell division, a process known as chromosomal instability (CIN) (2). CIN is a principal contributor to genetic heterogeneity in cancer (3) and is an important determinant of clinical prognosis and therapeutic resistance (4, 5). Over the past two decades, our understanding of the mechanisms that lead to CIN as well as our appreciation of its consequences on cellular viability and tumor evolution have grown considerably (4, 6). So has our recognition of the multitude of questions that remain unanswered.

The papers in this Research Topic broadly address recent advances in our understanding of CIN in cancer. They also illustrate the diverse experimental approaches and model organisms used in studying genomic instability. This topic is divided into two major categories: the first five papers address the genesis of CIN in cancer by summarizing the cell biological mechanisms that underlie chromosome missegregation. They also venture into the poorly understood area of the genetic basis of CIN, while developing an experimental model system amenable to high-throughput genetic analysis. The remaining papers address the consequences of imbalance in chromosome number on the cellular fitness and adaptation.

Multiple mechanisms have been shown to lead to CIN – in its numerical and structural forms. They include defects in the spindle assembly checkpoint (7), sister chromatid cohesion (8), the regulation of microtubule attachments to chromosomes at kinetochores (9, 10), centrosome duplication (11, 12), telomere maintenance (13), and pre-mitotic replication stress (14). Herein, German Pihan (15) reviews the regulation of the centrosome duplication cycle and how it is intricately synchronized with the cell division cycle. The complexity of this regulatory network might explain pervasiveness of centrosome dysfunction in human tumors, but it also provides multiple attractive pharmacological targets that have the potential to induce mitotic catastrophe. Yokoyama and Gruss (16) further discuss how chromosomes take on part of the responsibility to ensure the fidelity of their own segregation. Chromatin-associated factors – beyond the Ran GTPase – have now been shown necessary for a properly functioning mitotic spindle. Interestingly, many of these factors localize to the Nuclear Pore Complex (NPC), highlighting an incipient spatiotemporal relationship between the interphase nuclear structure and the mitotic spindle (17). Thus the process of faithful chromosome segregation starts well before the onset of mitosis.

While many of the cellular events that underlie CIN have now been uncovered, the genetic basis of chromosome missegregation and aneuploidy remains elusive. A growing number of genetic perturbations have been shown capable of inducing CIN in otherwise normal mammalian cell lines (6). Yet, it remains unknown whether these experimental conditions mimic naturally occurring genetic events that lead to CIN during tumor progression. Further complicating this matter is the self-propagating nature of CIN (18), which can mask initial instigating genetic triggers. Herein, Rao and Yamada (19) review the linear progression model of colon carcinogenesis from adenoma to carcinoma. They discuss how many of the sequential genetic events that occur during carcinogenesis have the potential to compromise the fidelity of chromosome segregation. More generally, Orr and Compton (20) discuss the intimate relationship governing CIN and known oncogenic pathways. Given what we now know, they argue that almost every major oncogenic pathway can be implicated in some manner in the genesis of CIN. Yet this relationship is almost certainly bidirectional as chromosome missegregation has been shown to generate downstream structural chromosomal damage, which can in turn independently activate oncogenic pathways (21–23). This complex relationship highlights the need for appropriate genetic models to better understand CIN. To this end, Salemi et al. (24) develop a chromosome segregation error correction assay, using the *Drosophila melanogaster* (Dm) S2 cells, that is amenable to high-throughput genetic screening. They substitute the Dm kinesin-5 protein, Klp61F, with its human ortholog, Eg5, thus acquiring the ability to purposely induce errors in microtubule attachments to chromosomes and subsequent chromosome missegregation through transient exposure to a small molecule inhibitor of Eg5 (25). These attachment errors occur in both normal and tumor cells alike (26), although cancer cells have been shown less efficient at correcting these errors (9). In this system, it would be feasible to screen for genes whose functions are to modulate the correction of microtubule attachment to chromosomes with the caveat that microtubule-associated proteins, particularly the kinesin family, may at times exhibit convergent evolution (27, 28) thus limiting direct comparative genetic analysis between Dm and humans.

The second part of this Research Topic addresses the consequences of CIN on cellular fitness in the context of tumor evolution. Roschke and Rozenblum (29) explore how CIN is tightly interconnected to other aspects of tumorigenesis such as DNA damage, loss of tumor suppressor genes, and gain of oncogenes. Importantly, they attempt to consolidate an apparent paradox in the field whereby the widespread prevalence of CIN in cancer stands in contrast to evidence showing that aneuploidy induces a proteotoxic stress response and reduces cellular fitness (30). They discuss the various pathways, which tumor cells utilize to cope with the cellular stressors involved with chromosome missegregation and they propose that tumor cells may balance the ability to rapidly proliferate with the need to generate sufficient diversity required for adaptation. One of the important adaptation mechanisms is the loss of the *p53* tumor suppressor pathway that normally limits the proliferation of aneuploid and tetraploid cells (18, 31). Although in this issue, Ohshima and Seyama (32) devise a method to derive tetraploid cells that appear to have an intact *p53*/*p21* signaling pathway that adequately responds to DNA damage, strongly suggesting the existence of alternative pathways that may confer tolerance to non-diploid karyotypes. Finally, Nicholson and Cimini (33) discuss the uni-directionality of CIN and aneuploidy. In most cases, it appears that once diploid cells become aneuploid, they also appear to become chromosomally stable. This positive feed-forward loop linking aneuploidy and CIN would constantly generate karyotypic heterogeneity that is shaped by natural selection. From this, the authors discuss how broad karyotypic patterns emerge that may shed light on defining karyotypic changes during tumor evolution.

## Conflict of Interest Statement

The authors declare that the research was conducted in the absence of any commercial or financial relationships that could be construed as a potential conflict of interest.
